# Performance of ASAP, LG2m, and GP73 for HCC Detection in Patients with Cirrhosis

**DOI:** 10.3390/cancers18162711

**Published:** 2026-08-21

**Authors:** Mohammad Jarrah, Mohammed Al-Hasan, Mariadelcarmen Yanez, Maria V. Yow, Eunice Amador, Diya Kar, Fouzia Ahmed, Nicole E. Rich, Sneha Deodhar, Jenna Bedrava, Claire Chen, Amit G. Singal

**Affiliations:** 1 Department of Internal Medicine, UT Southwestern Medical Center, Dallas, TX 75390, USA; 2 Abbott Diagnostics, Abbott Park, IL 60064, USA

**Keywords:** ASAP, hepatocellular carcinoma, biomarkers, surveillance

## Abstract

**Background:** The sensitivity of ultrasound plus alpha-fetoprotein (AFP) for early-stage hepatocellular carcinoma (HCC) detection remains suboptimal, prompting interest in blood-based biomarkers as an alternative strategy. The ASAP [age, sex, AFP, PIVKA-II] score has promising performance, although validation in contemporary Western populations is needed. **Methods:** We conducted a case–control study in which the cases were patients with HCC and the controls had cirrhosis without HCC. AFP, PIVKA-II, LG2m, and GP73 were measured using the Abbott Alinity platform, and ASAP scores were calculated. Sensitivity and specificity for early-stage HCC (BCLC stage 0/A) were compared between ASAP and ultrasound plus AFP using McNemar’s chi-square test. **Results:** Among 294 patients (median age of 61 years, 67.7% men), 50.7% had HCC (69.1% BCLC 0/A), and 49.3% had cirrhosis. The performance of individual biomarkers for early-stage HCC was moderate to poor (AUROC: AFP 0.71, PIVKA-II 0.73, LG2m 0.51, and GP73 0.53). ASAP, at a cutoff of ≥0.526, demonstrated a sensitivity of 88.3% for early-stage HCC, with specificity of 54.5%. Sensitivity was higher in men and viremic HCV, whereas specificity was higher in younger individuals, women, and Child–Pugh A cirrhosis. In subgroup analysis (n = 178), ASAP had comparable sensitivity to ultrasound plus AFP (85.5% vs. 90.3%; *p* = 0.51) but lower specificity (53.4% vs. 93.1%; *p* < 0.001). Using the Youden-optimized cutoff (≥0.804) improved specificity to 83.4% while maintaining a sensitivity of 68.0% for early-stage HCC. **Conclusions:** ASAP demonstrated high sensitivity for early-stage HCC detection but low specificity. These findings suggest a need for optimized cutoffs prior to implementation in clinical practice.

## Simple Summary

Hepatocellular carcinoma (HCC), the most common form of primary liver cancer, has better survival when detected at an early stage. Ultrasound plus alpha-fetoprotein (AFP), our current approach to screening, misses over one-third of cancers early, driving a growing interest in blood-based biomarkers. In this study, we evaluated the ASAP biomarker panel which demonstrated comparable sensitivity to ultrasound plus AFP for early-stage HCC, but its performance was hampered by low specificity (i.e., false-positive results). These findings highlight the promise of blood-based biomarkers for early HCC detection but underscore the need for further optimization prior to routine use in practice.

## 1. Introduction

Hepatocellular carcinoma (HCC) is the third leading cause of cancer-related death worldwide [1,2]. Survival is stage-dependent, with 5-year survival rates exceeding 60% for patients with early-stage HCC compared to only 20% for those diagnosed at more advanced stages [3]. This stark contrast in prognosis underscores the importance of effective surveillance strategies that optimize early detection [1,4]. Guidelines from the American Association for the Study of Liver Diseases (AASLD) and the European Association for the Study of the Liver (EASL) recommend semi-annual surveillance using abdominal ultrasound plus serum alpha-fetoprotein (AFP) in patients with cirrhosis or chronic hepatitis B virus (HBV) infection [1,4]. This strategy improves early-stage detection and overall survival; however, its real-world performance remains suboptimal, with a sensitivity of approximately 63% for early-stage HCC and global underutilization among at-risk populations [5,6,7]. Further, abdominal ultrasound is operator-dependent, and its performance is challenged by obesity and advanced liver disease [8,9,10]. These limitations may become more prevalent over time as HCC epidemiology shifts toward non-viral etiologies, a population in whom ultrasound quality is disproportionately poor [11].

This suboptimal performance has motivated considerable interest in alternative biomarker-based strategies as standalone or complementary surveillance tools [11]. In addition to AFP, protein induced by vitamin K absence or antagonist-II (PIVKA-II), also known as des-gamma-carboxy prothrombin (DCP), is a promising biomarker for HCC detection. In a previous study, PIVKA-II demonstrated an area under the receiver operating characteristic (AUROC) of 0.72 (95%CI 0.68–0.77) for early-stage HCC detection [12]. In addition to evaluating individual biomarkers as potential surveillance strategies, combining them with demographic variables has emerged as a strategy to improve diagnostic accuracy. One example is the multi-biomarker ASAP score, a classifier utilizing age, sex, AFP, and PIVKA-II [13]. A recent meta-analysis reported a pooled sensitivity and specificity of 72.1% and 83.3% [14]; however, most studies to date have been from Asian cohorts with majority HBV-infected individuals [15,16]. PIVKA-II and associated biomarker panels may have different performance characteristics in Western populations, given differences in the distributions of demographic features, liver disease etiologies, and degrees of liver dysfunction, underscoring a need for validation in contemporary cohorts from the United States or Europe.

Moreover, several other biomarkers have been evaluated for HCC detection, including Laminin γ2 Monomer (LG2m) and Golgi protein 73 (GP73). LG2m, a component of Laminin-332, induces tumor cell proliferation and is frequently expressed in several malignancies, including HCC [17,18]. GP73, a Golgi transmembrane protein expressed in epithelial cells, is upregulated in patients with chronic liver disease and those with HCC [19,20]. While potentially promising, most studies have assessed their performance alone or compared them with AFP alone, with few directly comparing their performance to ultrasound plus AFP [14].

Herein, we evaluated the diagnostic performance of the ASAP model and individual serum biomarkers, including PIVKA-II, LG2m, and GP73, for any-stage and early-stage HCC detection in a diverse cohort of patients in the United States.

## 2. Methods

### 2.1. Study Population

We conducted a retrospective case–control study using biorepository serum samples collected between December 2014 and October 2025 from patients at UT Southwestern Medical Center and Parkland Health in Dallas, Texas, USA. The cases were patients with newly diagnosed, treatment-naïve HCC, and the controls were patients with cirrhosis without evidence of HCC for at least 1 year of follow-up. HCC was diagnosed according to AASLD criteria, and cirrhosis was diagnosed by either histology, transient elastography, serum fibrosis markers, or a cirrhotic-appearing liver with portal hypertension on imaging [1]. The cases and controls were initially matched in a 1:1 ratio based on etiology, the year of blood collection (± 1 year) and the Child–Pugh score; however, subsequent exclusion of controls who developed HCC within 12 months led to small differences in etiology and the Child–Pugh score. Patients receiving warfarin or vitamin K supplementation were excluded due to their established interference with PIVKA-II measurement [21].

Baseline data extracted from the electronic medical record included age, sex, race and ethnicity, body mass index (BMI), and cirrhosis etiology and severity (the Child–Pugh score). Race and ethnicity were categorized as non-Hispanic White (White), non-Hispanic Black (Black), non-Hispanic Asian (Asian), and Hispanic. For patients with missing data for ethnicity (n = 3), race alone was used for classification. Liver disease etiologies were classified as viremic hepatitis C virus (HCV), post-sustained virologic response (SVR), HBV, alcohol-associated liver disease (ALD), metabolic dysfunction-associated steatotic liver disease (MASLD), or others. For HCC cases, tumor burden data were abstracted (number of liver lesions, maximum tumor diameter, vascular invasion, distant metastasis) from imaging reports. Early-stage HCC was defined as Barcelona Clinic Liver Cancer (BCLC) stage 0 or A. The study protocol (STU092013-010) was approved by the Institutional Review Board at UT Southwestern Medical Center.

### 2.2. Sample Processing and Biomarker Measurement

The blood samples were processed according to the Early Detection Research Network (EDRN) protocols, with all specimens processed within 4 h of collection and stored at −80 °C without interim thawing or re-freezing [22]. The samples were shipped to Abbott Diagnostics (Lake County, IL, USA) for analysis, where serum levels of AFP, PIVKA-II, LG2m, and GP73 were measured using the Abbott Alinity platform by staff blinded to HCC status. Abnormal values were defined using prespecified cutoffs: AFP ≥ 20 ng/mL, PIVKA-II ≥ 40 mAU/mL, and LG2m ≥ 30 pg/mL [1,12,23,24]. Given a lack of a predefined cutoff for GP73, it was evaluated at ≥189.26 ng/mL, as defined by Youden’s index in this cohort [24]. The ASAP score was calculated using a single cross-sectional AFP and PIVKA-II measurement at the time of blood collection, consistent with the developer’s methodology. The ASAP score was calculated in two steps. The logit was calculated using the following formula: y = −7.57711770 + 0.04666357 × [Age] − 0.57611693 × [Sex (1 for women, 0 for men)] + 0.42243533 × In[AFP] + 1.10518910 × ln[PIVKA-II]. Then the probability predicted by ASAP was calculated as: e^y^/(1 + e^y^) [13,16].

### 2.3. Ultrasound Assessment

Ultrasound results were obtained for patients who underwent imaging as part of routine clinical care by registered sonographers and interpreted by fellowship-trained radiologists [25]. An abnormal ultrasound was defined as detection of a liver lesion ≥1 cm. Ultrasound visualization quality was recorded when available per LI-RADS criteria: score A (no or minimal limitations), B (moderate limitations), and C (severe limitations) [26].

### 2.4. Statistical Analysis

Patient characteristics were summarized overall and compared among the cases and controls. Differences in patient characteristics were tested using the Mann–Whitney U test and chi-square tests. Diagnostic performance of individual biomarkers and ASAP was assessed using ROC curves, and AUROCs were compared using DeLong’s test. Sensitivity and specificity for any-stage and early-stage HCC were calculated for each surveillance strategy at the prespecified cutoffs, with 95% confidence intervals calculated using the Wilson score method. An ASAP score ≥ 0.526 was considered positive based on established cutoffs and recommendations [13,16]. We also performed two sensitivity analyses using: (1) Youden’s index to identify the optimal ASAP cutoff for this cohort and (2) a 3-tier classification in which ASAP is categorized using tertiles of HCC risk based on the following: low risk score (<0.33), medium risk (0.33–0.67), and high risk (≥0.67). This 3-tier classification has been primarily evaluated for risk stratification, although herein, we evaluated its potential utility for HCC surveillance.

In the subgroup of patients with both ultrasound and AFP results, differences in ASAP and ultrasound plus AFP performance were assessed using McNemar’s chi-square test using Edwards’ correction. The diagnostic odds ratios (DOR) of each test were calculated to quantify overall performance using the Haldane–Anscombe correction. Performance was assessed overall and across several subgroups, including age, sex, liver disease etiology, the Child–Pugh score, BMI, and tumor burden. All statistical analyses were performed using R version 4.4.2. A significance level of 0.05 was used for all hypothesis tests.

## 3. Results

### 3.1. Patient Characteristics

The characteristics of the 149 HCC cases and 145 cirrhosis controls are summarized in Table 1. The cases were older than the controls (median age of 63 vs. 57 years; *p* < 0.001). The cohort was predominantly 67.7% men and 42.5% White. Liver disease etiology was similar between the groups and included HCV (29.9% viremic HCV; 15.3% post-SVR), ALD (24.5%), and MASLD (16.0%). Most patients (59.9%) had Child–Pugh A cirrhosis, including 62.4% of cases and 57.2% of controls. The median BMI was 28.9 kg/m^2^, with 42.2% of patients classified as obese. Among the cases, 69.8% had unifocal tumors, with a median tumor diameter of 3.2 cm. The BCLC staging among the cases was distributed as follows: 16.8% stage 0, 52.3% stage A, 14.1% stage B, 14.1% stage C, and 2.7% stage D. Over half (59.1%) of the HCC cases were within the Milan criteria.

### 3.2. Individual Biomarkers

**Figure 1:**
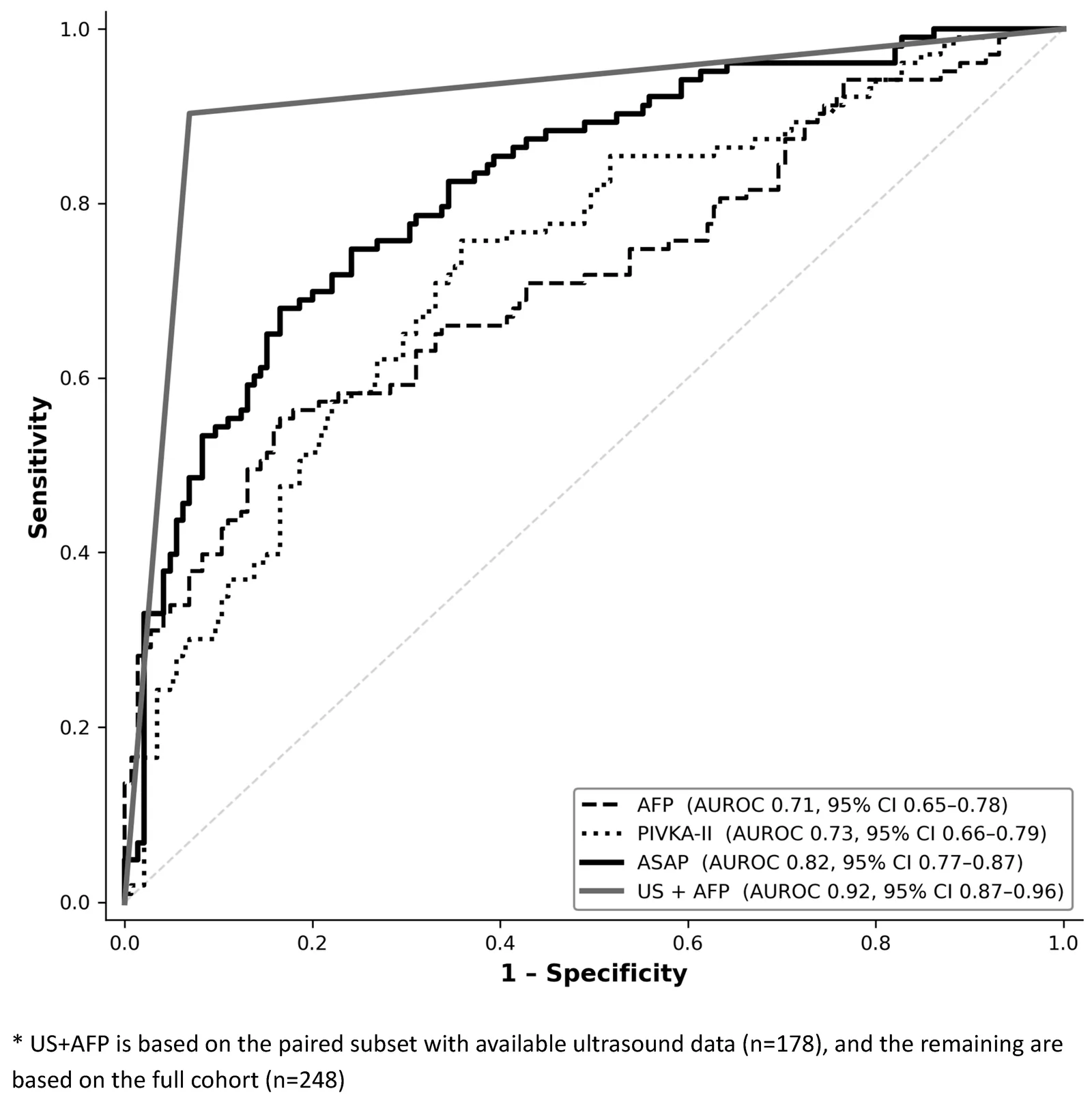
AFP, PIVKA-II, ASAP, and US + AFP * ROC curves for early-stage HCC Detection.

The cases had significantly higher median values of AFP (13.3 vs. 4.4 ng/mL; *p* < 0.001), PIVKA-II (399.2 vs. 49.7 mAU/mL; *p* < 0.001), and GP73 (191.7 vs. 155.8 ng/mL; *p* = 0.03) compared to the controls (Supplemental Figure S1). Conversely, LG2m levels were comparable between the cases and controls (60.0 vs. 59.3 pg/mL; *p* = 0.40). For early-stage HCC detection, AUROCs were 0.71 (95%CI 0.65–0.78) for AFP, 0.73 (95%CI 0.66–0.79) for PIVKA-II, 0.53 (95%CI 0.45–0.60) for GP73, and 0.51 (95%CI 0.44–0.59) for LG2m (Figure 1). At the prespecified cutoffs, AFP sensitivities were 43.6% for any-stage HCC and 34.0% for early-stage HCC, with a specificity of 94.5%. PIVKA-II demonstrated the highest sensitivity among individual biomarkers, with sensitivities of 89.3% for any-stage and 85.4% for early-stage HCC, but with a low specificity of 38.6%. GP73 sensitivities were 50.3% for any-stage and 44.7% for early-stage HCC, with a specificity of 62.1%. LG2m had sensitivities of 80.5% for any-stage and 80.6% for early-stage HCC, with a very low specificity of 18.6%.

The combination of AFP (≥20 ng/mL) and PIVKA-II (≥40 mAU/mL) demonstrated a sensitivity of 91.3% for early-stage HCC, with a specificity of 34.5%. Increasing the PIVKA-II cutoff to ≥100 mAU/mL improved the specificity to 70.3%, with a sensitivity of 62.1% for early-stage HCC. Adding LG2m (≥30 pg/mL) to ASAP showed a high sensitivity of 98.0% and 98.1% for any-stage and early-stage HCC, respectively, but with a very low specificity (13.8%). Adding GP73 (≥189.26 ng/mL) to ASAP demonstrated sensitivities of 95.3% and 94.2% for any-stage and early-stage HCC, respectively, with a specificity of 40.7%.

### 3.3. ASAP Score

We first evaluated the overall diagnostic accuracy of ASAP components. Age and sex alone demonstrated AUROCs of 0.70 (95% CI 0.64–0.77) and 0.43 (95%CI 0.35–0.50), respectively. Age and sex together achieved an AUROC of 0.74 (0.68–0.80). Adding AFP and PIVKA-II improved the AUROC to 0.79 (0.73–0.85). Finally evaluating the components using the ASAP score achieved an AUROC of 0.82 (0.77–0.88).

ASAP scores were significantly higher in the HCC cases compared to the controls (1.0 vs. 0.5; *p* <0.001). Diagnostic performance of ASAP is illustrated in Table 2. ASAP had an AUROC of 0.86 (95%CI 0.82–0.90), with 91.3% sensitivity and 54.5% specificity for any-stage HCC and an AUROC of 0.82 (95%CI 0.77–0.87), with 88.3% sensitivity and 54.5% specificity for early-stage HCC using the cutoff of 0.526. ASAP performance was generally consistent across subgroups, with a few exceptions. Early-stage HCC sensitivity was significantly higher in men than in women (94.7% vs. 71.4%; *p* = 0.001) and in viremic HCV patients vs. post-SVR patients (97.4% vs. 76.9%; *p* = 0.04). Conversely, specificity was significantly lower in older individuals aged ≥65 years compared to <65 years (37.0 vs. 58.5%; *p* = 0.04), men compared to women (46.4% vs. 65.6%; *p* = 0.02), and Child–Pugh B/C patients compared to Child–Pugh A (37.1% vs. 67.5%; *p* <0.001).

When applying the Youden-optimized cutoff (≥0.804), specificity improved to 83.4% while maintaining a sensitivity of 68.0% for early-stage HCC. Using the three-tier classification, 81.9% of the cases were classified as high risk of HCC, 15.4% as intermediate risk, and 2.7% as low risk. Among the controls, 30.3% were classified as high risk, 44.1% as intermediate risk, and 25.5% as low risk. Applying the high-tier threshold of ≥0.67, ASAP yielded a sensitivity of 75.7% for early-stage HCC, with a specificity of 69.7%. Applying the intermediate + high-tier threshold of ≥0.33 resulted in increased ASAP sensitivity for early-stage HCC of 96.1% but decreased specificity of 25.5%.

### 3.4. Abdominal Ultrasound with or Without Biomarkers

Ultrasound within 6 months of blood collection was available for early-stage performance in 178 patients (62 cases and 116 controls). The ultrasound demonstrated a sensitivity of 80.6% for early-stage HCC, with a specificity of 96.6%. When combined with AFP, the sensitivity increased to 90.3% for early-stage HCC, albeit with a small decrease in specificity to 93.1%, with an AUROC of 0.92 (0.87–0.96) (Figure 1). Using other biomarkers with ultrasound similarly resulted in higher sensitivity, albeit with larger decreases in specificity. PIVKA-II plus ultrasound had a sensitivity of 91.9% for early-stage HCC, with a specificity of 34.5%. Using a PIVKA cutoff of ≥100 mAU/mL improved specificity to 68.1% with comparable sensitivity. Adding LG2m to ultrasound yielded high sensitivity for early-stage detection but with a very low specificity (19.0%). GP73 with ultrasound demonstrated a sensitivity of 85.5% for early-stage HCC, with a specificity of 61.2%. Finally, adding ASAP to ultrasound yielded a sensitivity of 95.2% for early-stage HCC, with a specificity of 52.6%.

### 3.5. ASAP vs. Ultrasound Plus AFP

**Figure 2:**
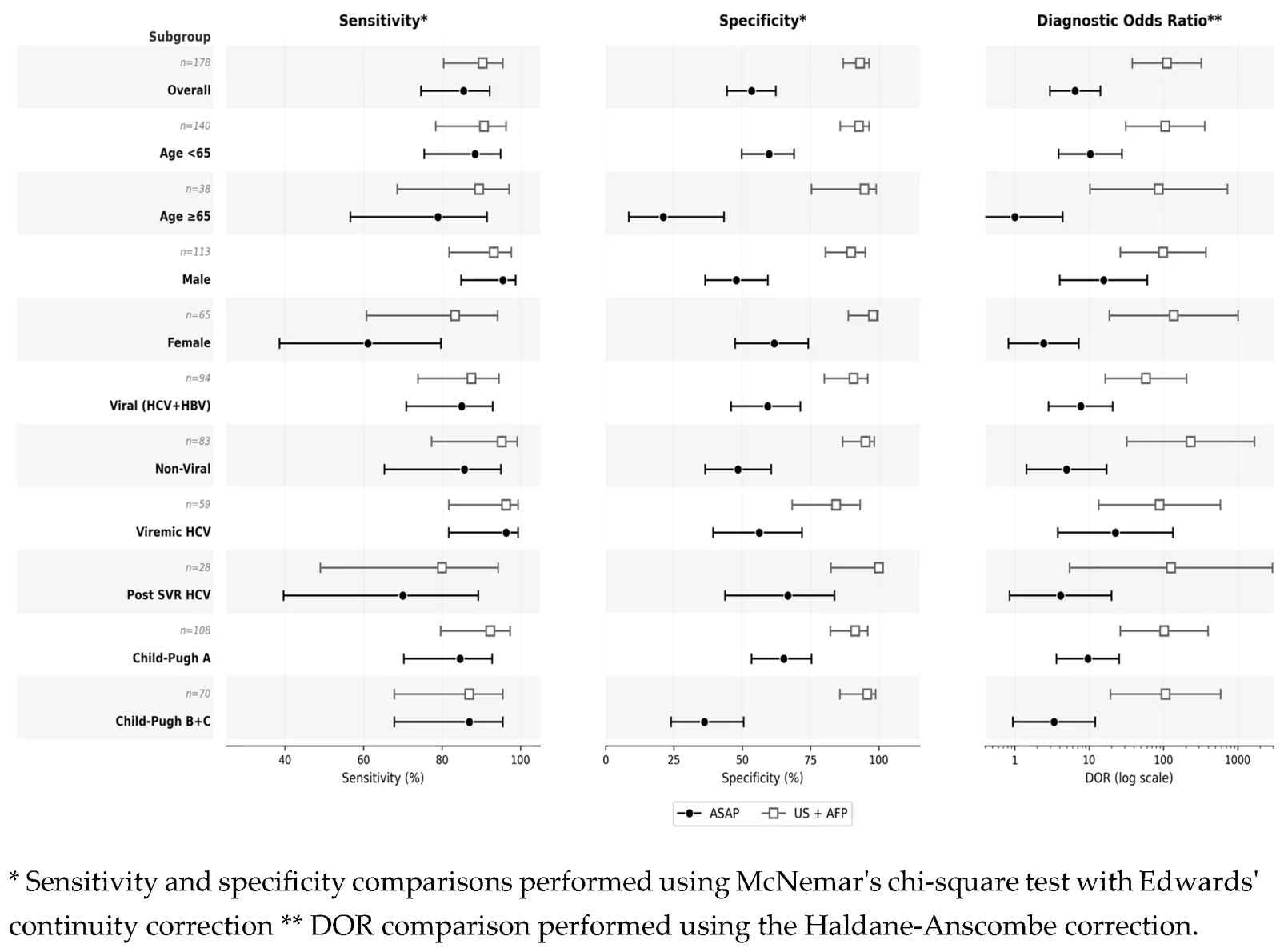
Performance of ASAP vs. ultrasound plus AFP for early-stage (BCLC 0/A) HCC detection, across patient subgroups.

We next evaluated performance among patients with both ASAP and ultrasound plus AFP data (n = 62 cases with early-stage HCC and 116 controls) (Figure 2). For early-stage HCC, ASAP had a similar sensitivity to that of ultrasound plus AFP (85.5% vs. 90.3%, *p* = 0.51) but with significantly lower specificity (53.4% vs. 93.1%; *p* < 0.001). The results in this subgroup were similar across all examined subgroups, including age, sex, etiology, Child–Pugh, and ultrasound visualization score categories. The DOR was higher for ultrasound plus AFP across most subgroups, except in men and patients with HCV infection, where performance was comparable.

Combining ultrasound and ASAP demonstrated higher sensitivity than ultrasound alone (95.2% vs. 80.6%; *p* = 0.008), but with significantly lower specificity (52.6% vs. 96.6%; *p* < 0.0001). The sensitivity of ultrasound plus ASAP was higher than ultrasound plus AFP (95.2% vs. 90.3%; *p* = 0.248), while specificity remained significantly lower (52.6% vs. 93.1%; *p* < 0.0001) (Supplemental Figure S2).

## 4. Discussion

In our case–control study of patients with diverse liver disease etiologies, PIVKA-II demonstrated the highest sensitivity for early-stage HCC among individual biomarkers but had low specificity, whereas LG2m and GP73 demonstrated poor performance for early-stage HCC detection. ASAP demonstrated high sensitivity for early-stage HCC, comparable to that of ultrasound plus AFP; however, its overall diagnostic performance was limited by low specificity. These findings provide important insight into the performance of the ASAP score, highlighting both its promise and limitations.

Given the limitations of ultrasound-based surveillance, there is growing interest in alternative imaging and blood-based biomarkers for HCC detection. Emerging imaging strategies such as CT scans, MRI, or abbreviated MRI-based surveillance are limited by logistical considerations such as higher costs, scheduling issues, radiation exposure, and lower adherence [27,28,29]. On the other hand, blood-based biomarker tools, such as ASAP, GAAD (gender, age, AFP, DCP), and GALAD (gender, age, AFP-L3, AFP, DCP) offer practical advantages such as improved adherence, objective interpretation, and integration into routine clinic visits [11]. Prior studies evaluating ASAP have been conducted in Asian cohorts with predominantly HBV, making our study an important addition to the literature by providing data in a diverse population encompassing viral and non-viral etiologies [13,15,16]. In the original development cohort, ASAP achieved 73.8% sensitivity for early-stage HCC at a fixed 90% specificity [13]. Subsequent validation studies have reported high specificities with AUROCs ranging from 0.88 to 0.90 [15,16]. A recent meta-analysis also demonstrated a pooled sensitivity of 72.1% and pooled specificity of 83.3% for ASAP, although most studies were conducted in Asia among HBV-predominant sample sets [14]. A study combining patients from Europe and Latin America demonstrated a low sensitivity of 45% with specificity of 91% for early-stage HCC detection [30]. In our study, the primary analysis demonstrated high sensitivity exceeding 80% but low specificity below 60%. When applying the Youden-optimized cutoff, specificity improved to >80% while maintaining an acceptable sensitivity > 65% for early-stage HCC. These data raise potential concerns regarding the generalizability of ASAP thresholds across different populations and etiologies and the importance of external validation studies examining transportability across different clinical contexts.

This low specificity of ASAP appears to be driven primarily by elevated PIVKA-II levels in controls. The median PIVKA-II level in our controls with cirrhosis (49.7 mAU/mL) exceeded the cutoff threshold used (40.0 mAU/mL), resulting in a substantial proportion of false-positive results. Indeed, several factors may contribute to this finding. PIVKA-II is an abnormal form of prothrombin resulting from impaired vitamin K-dependent carboxylation, and elevated levels have been reported in patients with vitamin K deficiency, ALD, elevated bilirubin, and patients with advanced chronic liver disease [31,32]. To address this, patients on warfarin or vitamin K supplementation were excluded from our study, and overt malnutrition was not clinically apparent in our cohort based on albumin, INR, and BMI values. The higher proportion of patients with Child–Pugh B cirrhosis in our cohort compared to that in prior studies (predominantly with viral hepatitis and compensated liver disease), as well as differences in age and sex distribution, may have contributed to the reduced specificity. Indeed, the specificity of ASAP was significantly higher in patients with Child–Pugh A cirrhosis than those with Child–Pugh B cirrhosis, younger versus older individuals, and women versus men. The lower specificity in older patients and men may also partly reflect the incorporation of age and sex into the ASAP formula, thereby increasing false-positive rates in these subgroups. The prior literature demonstrated elevated PIVKA-II levels in patients with Child–Pugh B and C cirrhosis, likely reflecting impaired vitamin K metabolism driven by advanced liver dysfunction as a contributing factor for this elevation [33]. Other than disease severity, ALD etiology also serves as another contributing factor to elevated PIVKA-II levels in patients with cirrhosis [34]. These findings highlight the limitation of biomarker thresholds derived from case–control studies in select populations and may not generalize well to heterogeneous real-world cohorts.

The specificity of 54.5% in our study using the recommended ASAP cutoff falls below the ≥80% threshold recommended by an expert consensus for HCC surveillance strategies [35]. This is concerning given the potential harms associated with false-positive results. Although physical harms from diagnostic follow-up evaluation are generally mild [36], consisting primarily of additional CT or MRI imaging rather than invasive procedures, the psychological and financial burdens of false-positive results can be clinically significant and should not be overlooked [37,38]. In our study, nearly half of cirrhosis controls were ASAP positive, triggering diagnostic evaluations. These findings underscore the importance of cutoff optimization for different populations and the potential need to refine thresholds to balance the inherent tradeoff between sensitivity and specificity. For example, the developers’ recommended ASAP cutoff of ≥0.526 may not be appropriate and generalizable to all populations. The Youden-optimized cutoff improved specificity to >80% while maintaining acceptable sensitivity for early-stage HCC. These data suggest that external validation and cutoff optimization in Western populations with predominant non-viral liver disease may be necessary to confirm its reproducibility and optimize the clinical utility of ASAP.

Our study also provides additional important data on LG2m and GP73 measured on the Abbott Alinity platform where LG2m showed no discriminatory ability to detect early-stage HCC (AUROC 0.51) and very low specificity. This contrasts with prior data, where LG2m demonstrated a sensitivity of 62.9% and specificity of 70.5% for HCC detection [23]. Whereas a meta-analysis previously reported a pooled sensitivity and specificity of 79% and 85% for GP73 in diagnosing HCC [39], it demonstrated modest performance for early-stage HCC detection in our study. Our data raise questions about their utility for HCC surveillance, and further evaluation in larger case–control studies is needed prior to consideration of phase 3 or phase 4 biomarker studies [40]. These findings suggest that GP73 may have a more relevant role as a marker of liver fibrosis severity rather than its use for HCC surveillance [41].

Our findings must be interpreted in the context of the study’s limitations. First, the case–control design is subject to selection and spectrum biases, which may overestimate test performance. Second, although the cases and controls were matched on etiology, the year of blood collection, and the Child–Pugh score, they differed in age and sex, which can impact biomarker performance. Third, ultrasound data were available for only a subset of patients, limiting the power of analyses. Ultrasound sensitivity also exceeded observed performance in the literature [6], which may reflect selection bias of HCC cases. Moreover, both institutions are academic centers with experienced radiologists, which may limit generalizability to other settings where ultrasound performance may be lower. Fourth, we used BCLC stage 0/A to define early-stage HCC, although other measures such as Milan Criteria, very early stage HCC < 2 cm, or BEACON Class 1 have been proposed to align with treatment allocation. Finally, our sample size limited power for some subgroup analyses of interest. Nonetheless, our study is important as it highlights the need for comprehensive evaluation of PIVKA-II-related panels to better understand their performance in contemporary populations.

## 5. Conclusions

In conclusion, ASAP demonstrated high sensitivity for early-stage HCC detection in a diverse Western cohort, although specificity was substantially lower than previously reported. These findings suggest that PIVKA-II-based biomarker panels may require optimization of thresholds, particularly in patients with advanced liver disease and non-viral etiologies, prior to widespread implementation.

## Figures and Tables

**Table 1:** Baseline characteristics of patients with HCC (cases) and cirrhosis (controls).

	HCC (n = 149)	Cirrhosis (n = 145)	*p*-Value ^δ^
Age, n (%)			
<65	92 (61.7%)	118 (81.4%)	<0.001
≥65	57 (38.3%)	27 (18.6%)	
Sex, n (%)			
Men	115 (77.2%)	84 (57.9%)	<0.001
Women	34 (22.8%)	61 (42.1%)	
Race/Ethnicity, n (%) *			
White	49 (32.9%)	76 (52.4%)	0.006
Hispanic	53 (35.6%)	44 (30.3%)	
Black	38 (25.5%)	22 (15.2%)	
Asian	7 (4.7%)	3 (2.1%)	
Etiology, n (%) **			
Viremic hepatitis C	51 (34.2%)	37 (25.5%)	0.01
Post-SVR hepatitis C	21 (14.1%)	24 (16.6%)	
Hepatitis B	7 (4.7%)	6 (4.1%)	
Alcohol-associated liver disease	37 (24.8%)	35 (24.1%)	
MASLD	21 (14.1%)	26 (17.9%)	
Other	5 (3.4%)	17 (11.7%)	
Child–Pugh Class, n (%)			
A	93 (62.4%)	83 (57.2%)	0.39
B	54 (36.2%)	57 (39.3%)	
C	2 (1.3%)	5 (3.4%)	
BMI Category, n (%)			
Underweight (BMI < 18.5)	4 (2.7%)	3 (2.1%)	0.76
Normal (BMI 18.5–24.9)	37 (24.8%)	29 (20%)	
Overweight (BMI 25–29.9)	49 (32.9%)	48 (33.1%)	
Obesity 1 (BMI 30–34.9)	34 (22.8%)	31 (21.4%)	
Obesity 2 (BMI 35–39.9)	14 (9.4%)	20 (13.8%)	
Obesity 3 (BMI ≥ 40)	11 (7.4%)	14 (9.7%)	
Tumor burden			
Number of lesions, median (IQR)	1 (1–1)		
Max tumor size (cm), median (IQR)	3.2 (2.2–5.5)		
Within Milan criteria, n (%)	88 (59.1%)		
BCLC Stage, n (%)			
0	25 (16.8%)		
A	78 (52.3%)		
B	21 (14.1%)		
C	21 (14.1%)		
D	4 (2.7%)		
AFP (ng/mL), median (IQR)	13.3 (5.1–125.8)	4.4 (2.9–7.5)	<0.001
PIVKA-II (mAU/mL), median (IQR)	399.2 (95.4–2530.0)	49.7 (31.7–133.5)	<0.001
GP73 (ng/mL), median (IQR)	191.7 (129.3–280.0)	155.8 (110.8–235.2)	0.03
LG2m (pg/mL), median (IQR)	60.0 (35.2–99.5)	59.3 (36.3–88.3)	0.40
ASAP Score, median (IQR)	1.0 (0.8–1.0)	0.5 (0.3–0.7)	<0.001

* A total of 2 cases had missing race information; ** 7 cases had no underlying liver disease and were excluded from etiology related analyses; ^δ^ differences in patient characteristics were tested using the Mann–Whitney U test and chi-square tests.

**Table 2:** Diagnostic performance of ultrasound and biomarkers for any-stage and early-stage HCC detection.

Test	AUROC * (95% CI)	Sensitivity (95% CI)	Specificity (95% CI)
Any-stage (n = 149 HCC, 145 Cirrhosis)			
US (n = 195)	0.87 (0.82–0.92)	77.2% (66.8–85.1)	96.6% (91.5–98.7)
US + AFP (n = 195)	0.89 (0.84–0.94)	84.8% (75.3–91.1)	93.1% (87.0–96.5)
ASAP (cutoff 0.526 − n = 294)	0.86 (0.82–0.90)	91.3% (85.6–94.8)	54.5% (46.4– 62.4)
ASAP − Youden ≥ 0.804 (n = 294)	0.86 (0.82–0.90)	76.5% (69.1–82.6)	83.4% (76.6–88.6)
Early-stage BCLC 0/A (n = 103 HCC, 145 Cirrhosis)			
US (n = 178)	0.89 (0.83–0.94)	80.6% (69.1–88.6)	96.6% (91.5–98.7)
US + AFP (n = 178)	0.92 (0.87–0.96)	90.3% (80.5–95.5)	93.1% (87.0–96.5)
ASAP (cutoff 0.526 − n = 248)	0.82 (0.77–0.87)	88.3% (80.7–93.2)	54.5% (46.4– 62.4)
ASAP − Youden ≥ 0.804 (n = 248)	0.82 (0.77–0.87)	68.0% (58.4–76.2)	83.4% (76.6–88.6)

* ASAP AUROC within the paired ultrasound subset: 0.83 (95% CI 0.77–0.89) for any-stage (n = 195) and 0.80 (95% CI 0.72–0.87) for early-stage (n = 178).

## Data Availability

Data supporting the findings of this study are available within the article and its Supplemental Materials. Other study materials and data related to the study are available from the corresponding author, upon reasonable request.

## Supplementary Materials

The following supporting information can be downloaded at: https://www.mdpi.com/article/10.3390/cancers18162711/s1, Figure S1. Serum biomarker levels in HCC cases and cirrhosis controls. Figure S2. Sensitivity and specificity for early-stage HCC detection.

## Abbreviations

HCC	hepatocellular carcinoma
AFP	alpha-fetoprotein
PIVKA-II	protein induced by vitamin K absence or antagonist-II
LG2m	laminin γ2 monomer GP73, Golgi protein 73
AUROC	area under the receiver operating characteristic curve
BCLC	Barcelona Clinic Liver Cancer
HCV	hepatitis C virus
SVR	sustained virologic response
DOR	diagnostic odds ratio
AASLD	American Association for the Study of Liver Diseases
EASL	European Association for the Study of the Liver
HBV	hepatitis B virus DCP, des-gamma-carboxy prothrombin
BMI	body mass index
ALD	alcohol-associated liver disease
MASLD	metabolic dysfunction-associated steatotic liver disease
EMR	electronic medical record
IRB	Institutional Review Board
EDRN	Early Detection Research Network
LI-RADS	Liver Imaging Reporting and Data System
MRI	magnetic resonance imaging
CT	computed tomography
INR	international normalized ratio
IQR	interquartile range
