# Supplementary material for: Performance of ASAP, LG2m, and GP73 for HCC Detection in Patients with Cirrhosis
Source: Cancers (Basel). 2026 Aug 21;18(16):2711. doi: 10.3390/cancers18162711 (PMC13511538; doi:10.3390/cancers18162711)
Supplement: Supplementary file 1 [file cancers-18-02711-s001.zip › cancers-4487995-supplementary.pdf]

Supplemental Figure S1. Serum biomarker levels in HCC cases and cirrhosis controls.

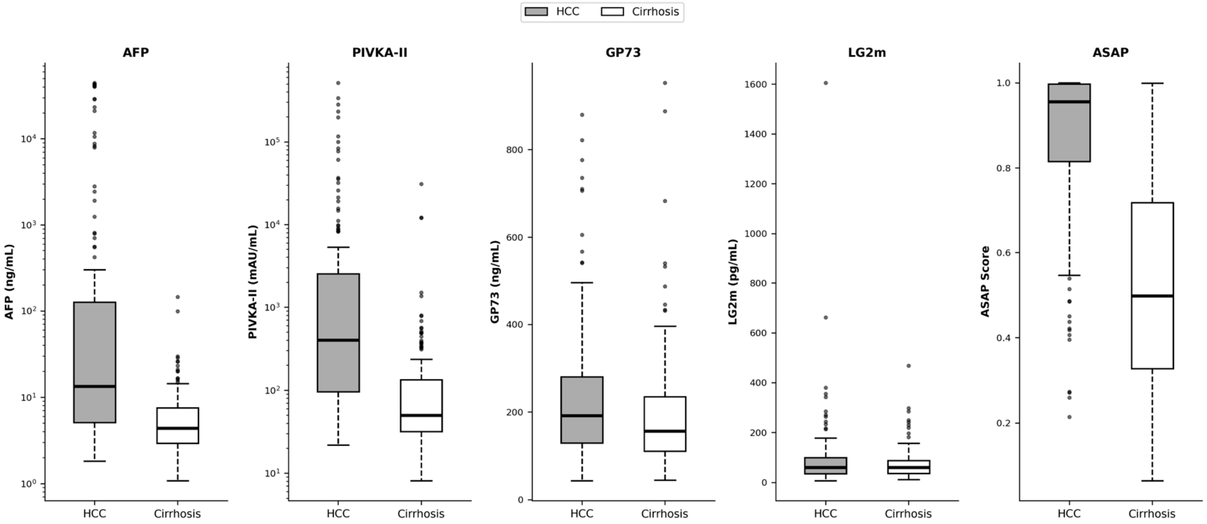

Supplemental Figure S2. Sensitivity and specificity for early-stage HCC detection.

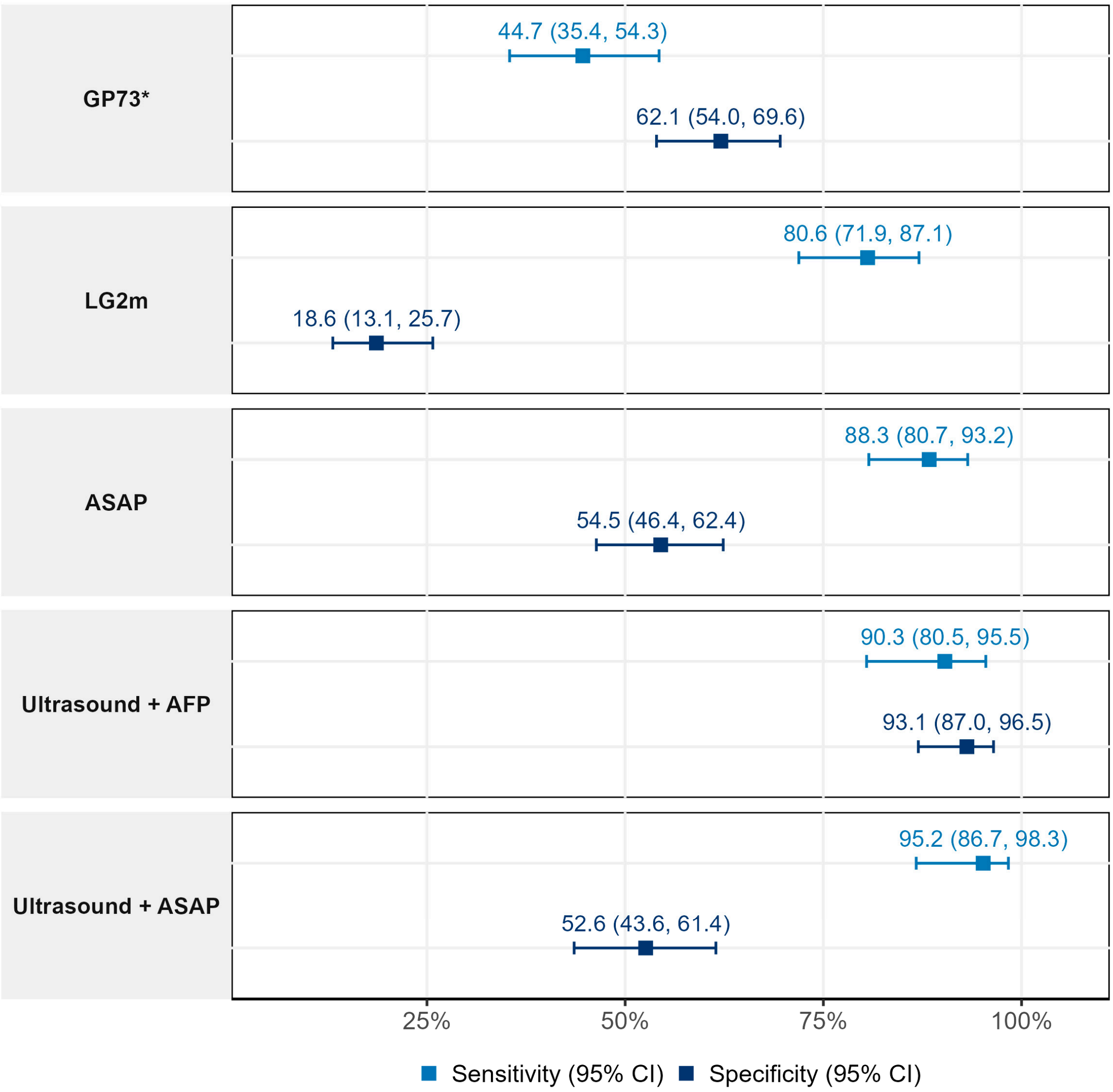

*GP73 performance was evaluated using the cutoff determined by Youden's index.
